# The Role of Actin Cytoskeleton in Memory Formation in Amygdala

**DOI:** 10.3389/fnmol.2016.00023

**Published:** 2016-03-31

**Authors:** Raphael Lamprecht

**Affiliations:** Sagol Department of Neurobiology, University of HaifaHaifa, Israel

**Keywords:** fear memory, drug memory, amygdala, actin cytoskeleton, spine morphology, glutamate receptors

## Abstract

The central, lateral and basolateral amygdala (BLA) nuclei are essential for the formation of long-term memories including emotional and drug-related memories. Studying cellular and molecular mechanisms of memory in amygdala may lead to better understanding of how memory is formed and of fear and addiction-related disorders. A challenge is to identify molecules activated by learning that subserve cellular changes needed for memory formation and maintenance in amygdala. Recent studies show that activation of synaptic receptors during fear and drug-related learning leads to alteration in actin cytoskeleton dynamics and structure in amygdala. Such changes in actin cytoskeleton in amygdala are essential for fear and drug-related memories formation. Moreover, the actin cytoskeleton subserves, after learning, changes in neuronal morphogenesis and glutamate receptors trafficking in amygdala. These cellular events are involved in fear and drug-related memories formation. Actin polymerization is also needed for the maintenance of drug-associated memories in amygdala. Thus, the actin cytoskeleton is a key mediator between receptor activation during learning and cellular changes subserving long-term memory (LTM) in amygdala. The actin cytoskeleton may serve as a target for pharmacological treatment of fear memory associated with fear and anxiety disorders and drug addiction to prevent the debilitating consequences of these diseases.

## Introduction

This review describes and discusses the mechanisms whereby actin cytoskeleton in amygdala mediates fear and drug-associated memory formation. In fear conditioning (FC) a conditioned stimulus (CS; e.g., innocuous tone or a context) is associatively paired with an aversive unconditioned stimulus (US; e.g., a mild footshock; LeDoux, 2000; Davis and Whalen, 2001; Schafe et al., 2001; Sah et al., 2003; Rodrigues et al., 2004; Maren, 2005; Johansen et al., 2011). FC leads to long-term memory (LTM) of the CS and the CS elicits fear responses when it is subsequently encountered. The hippocampus is involved in contextual FC memory (Kim and Fanselow, 1992; Phillips and LeDoux, 1992). In auditory FC information about the CS and US is transferred to the lateral nucleus of the amygdala (LA) from thalamus and cortex and the CS or US leads to responses in LA cells and some cells are activated by both stimuli (e.g., LeDoux, 2000). FC leads to changes in both excitatory and inhibitory responses with the net enhancement of auditory and footshock responses and promotion of CS-US association. For example, auditory stimulus leads to PV+ interneurons excitation and indirectly, via SOM+ interneurons, disinhibition of dendrites of basolateral amygdala (BLA) principal neurons. Aversive footshock leads to both PV+ and SOM+ interneurons inhibition, which increase postsynaptic footshock responses (Wolff et al., 2014). GABA transmission in the amygdala also contributes to extinction of fear memory (Lin H.C. et al., 2009). Inactivation of the LA during acquisition impairs learning (e.g., LeDoux et al., 1990; Helmstetter and Bellgowan, 1994; Muller et al., 1997; Fanselow and LeDoux, 1999; Wilensky et al., 1999; Nader et al., 2001), and neural activity in LA is altered by fear learning (e.g., Quirk et al., 1995, 1997; Collins and Paré, 2000; Repa et al., 2001). LA projects to other amygdala nuclei including the central nucleus of the amygdala (CE). The CE is an output nucleus of the amygdala projecting to brain areas involved in fear responses (e.g., LeDoux, 2000). The CE is also needed for fear memory formation and fear learning changes neural activity in CE (Nader et al., 2001; Wilensky et al., 2006; Ciocchi et al., 2010; Haubensak et al., 2010). BLA also transfers information to additional brain areas to affect fear memory. For example, GABAergic transmission in BLA modulates the structural changes in hippocampus associated with the influence of stress on fear memory (Giachero et al., 2015).

Amygdala is also involved in formation of drug-related memories such as formed in drug conditioned place preference (CPP) and conditioned place aversion (CPA). In CPP an associative memory is formed between environmental cues and the rewarding affective state produced by the drug treatment leading to the preference of this environment. CPP is mediated by a circuit that includes the BLA (e.g., Everitt et al., 1991; Hiroi and White, 1991; Brown and Fibiger, 1993; Hsu et al., 2002; Fuchs et al., 2005) and the hippocampus (e.g., Zarrindast et al., 2007). In CPA an association is made between drug negative affective consequences of withdrawal and a particular environment, leading to avoidance of the paired environment. CPA also depends on the amygdala including the central amygdala (e.g., Watanabe et al., 2002, 2003).

These observations beg the question: what are the molecular mechanisms that lead to memory formation in the amygdala? In this review evidence is provided and discussed showing that the actin cytoskeleton serves as a mediator between synaptic events that occur during fear and drug-related learning and cellular events underlying memory formation. Moreover, actin cytoskeleton is also needed for the maintenance of certain LTMs in amygdala.

## Modulation of Actin Cytoskeleton in Amygdala by Learning

Actin is found in cells as a monomer (G-actin) or after G-actin interactions as a polymer (F-actin). Actin cytoskeleton polymerization in amygdala during and shortly after FC and CPA learning is needed for memory formation as inhibition of actin polymerization at these time points impairs LTM. Microinfusion of cytochalasin D, an actin polymerization inhibitor, into rat LA immediately before or after FC training impaired fear LTM but not short-term fear memory (STM; Mantzur et al., 2009; Gavin et al., 2011). Rehberg et al. (2010) showed that microinjection of the actin depolymerization inhibitor phalloidin into BLA 6 h after FC impaired auditory fear memory. Cytochalasin D infused into the BLA impaired the return of fear after reconditioning at the last extinction session showing that polymerization of actin is required for reconditioning (Motanis and Maroun, 2012).

Actin cytoskeleton polymerization is also involved in conditioned morphine withdrawal (CMW) memory a CPA paradigm. CMW learning induced rearrangements in actin cytoskeleton (increase in the ratio of F-actin to G-actin) in the amygdala (Hou et al., 2009). Moreover, infusion into amygdala of latrunculin A (LatA), an inhibitor of actin polymerization, before CMW training attenuated CPA significantly (Hou et al., 2009).

Several findings point at the glutamate receptors as central effectors of actin cytoskeleton dynamics in learning. NMDA receptors (NMDARs) are essential for FC memory acquisition (Miserendino et al., 1990; Maren et al., 1996; Gewirtz and Davis, 1997; Rodrigues et al., 2001). It is not known whether NMDAR activation in LA during FC learning leads to actin polymerization. However, several studies suggest that NMDAR may be involved in actin polymerization in LA. Actin dynamics in spines are regulated by activation of either AMPA or NMDA subtype glutamate receptors (Fischer et al., 2000). In particular it was shown in primary hippocampal neurons that profilin is targeted to spine heads when postsynaptic NMDARs are activated (Ackermann and Matus, 2003). Profilin regulates actin polymerization by binding to G-actin enhancing the ADP-ATP exchange and thereby increasing the pool of cellular ATP-actin (Witke, 2004). Similarly, FC induced the translocation of profilin into LA dendritic spines in rats (Lamprecht et al., 2006). Dentritic spines in LA receive excitatory glutamatergic inputs from cells located in the auditory thalamus and auditory cortex brain areas needed for fear memory formation (Farb et al., 1995; Farb and LeDoux, 1997; Radley et al., 2007). Cumulatively, these findings suggest that glutamate receptors, in particular NMDAR, activation during FC learning lead to regulation of actin cytoskeleton dynamics, through profilin, in amygdala.

More direct evidence for the role of NMDAR in alteration of actin cytoskeleton dynamics after learning was shown using the CMW paradigm. It was shown that actin polymerization in the amygdala induced by CMW depends on NMDAR activation. Infusion of D-AP5, an NMDAR antagonist, into the rat amygdala 30 min or 10 min before CMW blocked actin polymerization induced by CMW (Liu et al., 2012). D-AP5 and MK-801 (another NMDAR antagonist) suppress the formation of morphine withdrawal-induced CPA (Watanabe et al., 2002).

Taken together, the above observations indicate that learning leads to alteration in actin cytoskeleton polymerization through glutamate receptors. Such changes in actin polymerization are essential for memory formation in amygdala. What are the molecular and cellular events that are subserved by actin cytoskeleton needed for memory formation in amygdala?

## Actin Cytoskeleton Affects Cellular Processes in Amygdala Subserving Memory Formation

Evidence suggests that memory is subserved by alterations in neuronal morphology and connectivity and synaptic transmission leading to changes in synaptic efficacy (Konorski, 1948; Hebb, 1949; Bliss and Collingridge, 1993; Martin et al., 2000; Kandel, 2001; Lamprecht and LeDoux, 2004). Memory formation in amygdala involves changes in neuronal morphogenesis, particularly of dendritic spines, and in glutamatergic synaptic transmission.

### Actin in Neuronal Morphogenesis

Most excitatory synapses in the brain terminate on dendritic spines. Spine morphology affects its functions, such as local voltage amplification, biochemical compartmentalization and postsynaptic glutamate sensitivity, and changes in spine morphology are involved in synaptic plasticity (e.g., Nimchinsky et al., 2002; Lamprecht and LeDoux, 2004; Newpher and Ehlers, 2009; Nishiyama and Yasuda, 2015). For example, in BLA principal neurons coupling between the spine and parent dendrite is determined by spine neck length with better calcium diffusion in spines with short neck (Power and Sah, 2014). Actin cytoskeleton is intimately involved in regulating spine morphology (e.g., Hotulainen and Hoogenraad, 2010). FC learning modulates the number of dendritic spines and their morphology. For example, auditory FC leads to an increase in spinophilin-labeled dendritic spines in the LA (Radley et al., 2006). Spinophilin, a F-actin interacting protein, is enriched in dendritic spines (Muly et al., 2004; Ouimet et al., 2004) and is implicated in regulation of spine morphology and density, synaptic plasticity and neuronal migration (Sarrouilhe et al., 2006). In addition, an increase in postsynaptic density (PSD) area and decrease in head volume of smooth endoplasmic reticulum (sER)-free spines is observed after FC (Ostroff et al., 2010). Actin cytoskeleton may be involved in regulating changes in spine morphology after FC. For example, auditory FC induces the movement of profilin into dendritic spines in rat LA and spines containing profilin have longer PSDs (Lamprecht et al., 2006). These results suggest that profilin and actin contribute to the enlargement in dendritic spines in LA after FC.

Changes in synapses and spine properties are also associated with drug CPP memory. Excitatory synapse number increases in the BLA with amphetamine CPP (Rademacher et al., 2010). Spine density in basolateral amygdala complex (BLC; lateral and basolateral) of METH-CPP trained animals increased with training. Such an increase in spine number depends on actin cytoskeleton as LatA microinjection into BLC 2 days after CPP training reduced spine density in METH-paired animals (Young et al., 2014). These findings show that learning leads to changes in spine properties and number in amygdala and that actin cytoskeleton is intimately involved in these processes.

### Actin in Glutamate Receptors Trafficking

Synaptic efficacy changes in amygdala could be supported also by alterations in glutamate receptors number in synapses. It was shown that FC leads to AMPA receptors (AMPARs) insertion into LA synapses an event needed for FC memory formation (Rumpel et al., 2005; Yeh et al., 2006; Nedelescu et al., 2010). The insertion and removal of AMPARs are mediated by AMPARs interacting proteins (e.g., Malinow and Malenka, 2002; Esteban, 2008; Anggono and Huganir, 2012) some exerting their function via interaction with the actin cytoskeleton (Hanley, 2014). The GluA1 subunit of AMPAR interacts directly with the F-actin-associated proteins 4.1N and 4.1G (Shen et al., 2000). GluA1 lacking the 4.1G/N binding site, showed decreased surface expression and this mutation occluded the effects caused by treatment with latrunculin (Shen et al., 2000). 4.1N was also shown to be needed for activity-dependent GluA1 plasma membrane insertion an important step in the synaptic delivery of AMPARs induced by stimuli leading to synaptic plasticity (Lin D.T. et al., 2009). Protein kinase C (PKC) phosphorylation of the serine 816 (S816) and S818 residues of GluA1 enhanced 4.1N binding to GluA1 and its insertion. Surface expression of GluA1 and the expression of long-term potentiation (LTP) are reduced after interfering with 4.1N-dependent GluA1 membrane insertion (Lin D.T. et al., 2009). PKC phosphorylation site (S818) of GluA1 is phosphorylated during LTP and is needed for its trafficking into the synapse and for LTP in hippocampus (Boehm et al., 2006). In addition it was shown that surface expression of GluA4 subunit is dependent on an interaction between its C-terminal domain and 4.1 protein (Coleman et al., 2003). Of note however is the finding that hippocampal CA1 basal synaptic transmission and LTP are unaffected in mutant mice expressing only 22% of 4.1N levels and lacking entirely 4.1G the Wozny et al. (2009).

To study the roles of 4.1N binding domain of GluA1 in fear memory formation in LA a peptide MPR(DD) comprising of a GluA1 MPR site with phospho-mimicking aspartates instead of serines (S816, S818) was used (Boehm et al., 2006; Mitsushima et al., 2011). Expression of MPR(DD) protein fragment prevents synaptic delivery of endogenous GluA1-containing AMPARs (e.g., Mitsushima et al., 2011) presumably by interacting with proteins such as 4.1N required for their synaptic incorporation. To study the roles of GluA4 4.1 interaction domain in amygdala in fear memory formation a MPR(AA) protein fragment was used. MPR(AA) derived from GluA4 interacts with proteins, including 4.1, required for GluA4 trafficking into the synapse (Coleman et al., 2003). Replacing alanines 816 and 818 to serines in the MPR of GluA4 abolished GluA4 trafficking into the synapse (Boehm et al., 2006). Microinjection of (MPR-DD) into LA before FC impaired both LTM and STM (Ganea et al., 2015). This finding is consistent with the study showing that blocking GluA1-containing AMPAR insertion in LA impaired both FC STM and LTM (Rumpel et al., 2005). Microinjection of MPR(AA) into LA before FC impaired fear LTM but not STM (Ganea et al., 2015). These observations suggest that AMPAR insertion into LA neuronal membrane is needed for FC and may be mediated by interaction of AMPAR with the actin binding protein 4.1N/G.

Regulation of AMPAR at the synapses by actin may also be needed for CPA. The expression of the cytoskeleton-associated protein Arc/Arg3.1 is increased and accumulates at synapses in the amygdala in response to CMW (Liu et al., 2012). Furthermore, knockdown of amygdalar Arc/Arg3.1 blocked CPA induced by CMW and impaired CMW-induced AMPAR endocytosis. AMPAR internalization is needed for CPA as intra-amygdala injection of Tat-GluR23Y prevented both the formation of CPA and the endocytosis of AMPARs induced by CMW. Tat-GluR23Y is a cell permeable peptide that competitively disrupts the GluR2 clathrin adaptor protein interaction preventing AMPAR endocytosis and LTD and affects memory (e.g., Brebner et al., 2006; Yu et al., 2008; Lin et al., 2010; Lopez et al., 2015). Importantly, the increase of Arc/Arg3.1 protein expression at synapses was disrupted by blockade of actin polymerization. Thus, Arc/Arg3.1 translocates to the synapses through actin polymerization, and regulates synaptic AMPAR endocytosis needed for CPA. Interestingly, Arc is also involved in fear memory formation in amygdala (Ploski et al., 2008; Nakayama et al., 2016).

Cumulatively, the aforementioned observations suggest that actin cytoskeleton may be involved in AMPAR trafficking needed for memory formation in amygdala.

## Are Changes in Actin Polymerization Needed for the Maintenance of Memory?

The observations above show that actin cytoskeleton modified during and shortly after learning, is needed for memory acquisition and consolidation and for alterations in neuronal morphology and glutamate signaling involved in memory formation. Is actin cytoskeleton also needed later in amygdala for maintenance of LTM? A recent study shows that intra BLC injection of LatA 2 days after training for CPP, where animals trained to associate the rewarding effects of METH with the environmental context, impaired CPP memory tested 15 min or 24 h afterwards (Young et al., 2014). LatA also disrupted the maintenance of context-induced reinstatement of METH seeking an instrumental learning of a contextual memory. These results show that actin cytoskeleton polymerization is needed for maintenance of CPP memory. It may not be the case for all amygdala dependent type of memories as fear memory is not impaired when cytochalasin D is injected into LA before fear memory retrieval suggesting that in FC actin cytoskeleton is needed for memory consolidation but not maintenance (Mantzur et al., 2009). However, this subject needs further scrutiny with additional actin cytoskeleton inhibitors (affecting different actin cytoskeletal properties), time of inhibitors injection and behavioral training parameters.

## Conclusions and Discussion

The above studies lead to several additional insights: (1) Although actin is needed for basic neuronal functions including synaptic transmission and morphogenesis interfering with functions of actin cytoskeleton in amygdala has no effect on fear memory acquisition but specifically on its consolidation. A tenable hypothesis is that actin cytoskeleton affects neuronal functions in amygdala needed specifically for fear memory consolidation; (2) The actin cytoskeleton in amygdala is involved in maintenance of certain memories. Moreover, the results indicate that perpetually proper actin cytoskeleton dynamic is part of a mechanism required for maintaining memory long after it has been consolidated; and (3) Interruption with actin cytoskeleton mediated functions in amygdala is sufficient for impairing memory formation. Other neuronal molecular events shown to be needed for the formation of memory such as protein synthesis could be mediated by actin cytoskeleton or are required for memory formation in addition to actin cytoskeleton at same or different time points.

## Actin Cytoskeleton in Amygdala Mediates Between Learning and Memory Formation

The observations above indicate that actin cytoskeleton mediates between learning and memory formation in amygdala. A sequence of events involving the actin cytoskeleton in amygdala leading to memory formation and maintenance is suggested. Learning leads to the activation of receptors and channels in the synapse above a certain level (for FC, see Blair et al., 2001) and subsequently to the activation of intracellular molecular pathways and alterations of actin cytoskeleton dynamics, structure and interactions. These alterations in actin cytoskeleton underlie central cellular events including changes of neurotransmitter receptors level at the synapse and in neuronal morphology. Such alterations can change synaptic efficacy through alteration in synaptic transmission and are believed to be essential for alterations in connectivity between neurons which constitute the changes in neuronal circuits subserving memory storage in amygdala. Enduring alterations in actin cytoskeleton and its dynamics supporting these cellular events after learning are also needed for maintenance of memory.

## Future Research

Much evidence indicates that the actin cytoskeleton is needed for the formation of memory in amygdala. However, central questions remain to be answered. For instance, are the alterations in cellular morphology such as changes in spine number and structure known to be subserved by actin cytoskeleton required for memory formation in amygdala? Do changes in synaptic transmission regulated by actin cytoskeleton required for memory formation? Studies aimed to answer such questions will provide vital insights into the roles of actin cytoskeleton in formation of memory and its maintenance in amygdala. Furthermore, future studies are needed to evaluate whether the actin cytoskeleton may serve as a target for pharmacological treatment of fear memory associated with fear and anxiety disorders and of drug addiction to prevent the debilitating consequences of these diseases.

## Author Contributions

RL wrote the manuscript. The author confirms being the sole contributor of this work and approved it for publication.

## Conflict of Interest Statement

The author declares that the research was conducted in the absence of any commercial or financial relationships that could be construed as a potential conflict of interest.
